# Modulation of cellular senescence in psoriatic arthritis: exploring the potential impact of bDMARDs on telomere length, mtDNA copy number, and oxidative damage

**DOI:** 10.1007/s10238-025-01952-0

**Published:** 2025-11-18

**Authors:** Giada De Benedittis, Eneida Cela, Chiara Morgante, Andrea Latini, Arianna D’Antonio, Mauro Fatica, Giulia Mori, Paola Conigliaro, Cinzia Ciccacci, Giuseppe Novelli, Maria Sole Chimenti, Paola Borgiani

**Affiliations:** 1 Department of Biomedicine and Prevention, Section of Genetics, University of Rome “Tor Vergata”, 00133 Rome, Italy; 2 Rheumatology, Allergology and Clinical Immunology, Department of System Medicine, University of Rome “Tor Vergata”, 00133 Rome, Italy; 3 UniCamillus–Saint Camillus International University of Health Sciences, 00131 Rome, Italy; 4 Giovanni Lorenzini Medical Foundation, 20129 Milan, Italy

**Keywords:** Psoriatic Arthritis, b/tsDMARDs, Telomere length, mtDNA copy numbers

## Abstract

Inflammation and cellular senescence are two interconnected biological processes critical for several chronic diseases. Pro-inflammatory factors promote the accumulation of reactive oxygen species, which synergistically accelerate the deterioration process. This creates a feedback loop that exacerbates telomere attrition and mitochondrial dysfunction, hallmarks of cellular senescence. Conventional therapies have been shown to influence oxidative stress and consequently cellular senescence, while the impact of biologic and targeted synthetic disease-modifying antirheumatic drugs (b/tsDMARDs) is poorly investigated. Our aim was to explore biomarkers of cellular senescence in a population of psoriatic arthritis (PsA) patients treated with different b/tsDMARDs; we evaluated telomere length (TL), mtDNA copy numbers and oxidative damage at the beginning of therapy and after 12 months. We enrolled 50 PsA patients starting a b/tsDMARDs treatment and monitored the response to treatment for 12 months, based on the Disease Activity Index for PsA score, to identify subgroups of patients: responders and non-responders. We collected a blood sample for each patient at the beginning of therapy and after 12 months. In addition, we collected blood samples of 34 age- and sex-matched controls (CTRLs). We evaluated the cellular senescence biomarkers by qPCR. PsA patients at T0 showed a lower TL and fewer mtDNA copy numbers with respect to CTRLs (*P* < 0.001). Moreover, oxidative telomeric and mitochondrial damage seems to correlate positively with BMI (*P* < 0.05). We performed a combined ROC curve analysis to evaluate their ability to discriminate the two groups, showing an AUC of 0.828 with 59.2% sensitivity and 93.7% specificity. After 12 months of treatment monitoring, we classified 36 patients as responder and observed a TL significantly longer compared to T0 (*P* < 0.001), reaching comparable values to those of the CTRLs group. After patients’ stratification based on the class of drugs, the result is confirmed in patients treated with TNFαi and IL17Ai. Our study provides novel insights for the molecular mechanism underlying PsA pathogenesis, highlighting the potential use of TL and mtDNA copy numbers as biomarkers for assessing cellular senescence in PsA. Moreover, our results also suggested that b/tsDMARDs may decelerate cellular senescence in PsA by preserving telomere length.

## Introduction

Inflammation and cellular senescence are two interconnected biological processes that play a critical role in aging and several chronic diseases [1]. It is known that pro-inflammatory factors promote senescence by stimulating the accumulation of reactive oxygen species (ROS), which, in turn, synergistically accelerate the deterioration process [2]. This creates a feedback loop that exacerbates telomere attrition and mitochondrial dysfunction, two of the main hallmarks of cellular senescence [3]. Chronic inflammation is a characteristic feature of autoimmune rheumatologic diseases, such as psoriatic arthritis (PsA). In these conditions, immune cells like T-lymphocytes and dendritic cells release pro-inflammatory cytokines, including TNFα and IL17A, contributing to persistent inflammation in both the joints and the skin of these patients [4, 5]. This inflammatory environment not only contributes to tissue damage but is also closely linked to cellular aging mechanisms [6].

Rheumatic inflammatory diseases are known to be characterized by telomere dysfunction [7]. Telomeres are tandem repeats of the nucleotide sequence “TTAGGG” associated with specific proteins that protect chromosome integrity [8]. Numerous studies have shown that T cells of these patients exhibit telomere shortening and reduced telomerase activity contributing to the immune dysregulation [9, 10]. Furthermore, oxidative stress (OS), prevalent in these patients, likely exacerbates telomere dysfunction, as telomere sequences are rich in guanine which is vulnerable to ROS-induced damage [11, 12]. Mitochondrial dysfunction also plays a significant role in the pathogenesis of rheumatologic disease, influencing innate immunity through redox-sensitive inflammatory pathways [13]. Mitochondria have their own genome, mitochondrial DNA (mtDNA), a double‑stranded molecule present in multiple copies per cell. The mtDNA copy number is considered an indicator of mitochondrial health and function, as it reflects the number of mitochondria in the cell [14]. Several studies have reported a reduced mtDNA copy number in leukocytes of patients with rheumatic inflammatory diseases compared to healthy controls [15].

Some conventional drugs used in the treatment of rheumatic inflammatory diseases, such as hydroxychloroquine, have shown direct effects in reducing OS [16]. Currently, biologic and targeted synthetic disease-modifying antirheumatic drugs (b/tsDMARDs) are commonly prescribed by rheumatologist because of their efficacy in reducing the activity of pathogenic cytokines and alleviating clinical symptoms [17]. Therefore, the ability of b/tsDMARDs to reduce the inflammation status could indirectly affect the levels of OS [18] and, consequently, cellular senescence. However, to date, no studies have evaluated whether and how these treatments could correlate with cellular senescence markers.

Based on this knowledge, our aim was to investigate biomarkers of cellular senescence in a population of PsA patients treated with different b/tsDMARDs drugs; in particular, we evaluated the telomere length (TL), the mtDNA copy number, and the oxidative damage at the beginning of therapy (T0) and after 12 months (T12).

## Material and methods

### Patients recruitment

We enrolled 50 PsA patients starting a b/tsDMARDs treatment (TNFα inhibitor [TNFαi], IL17A inhibitor [IL17Ai], or Janus kinase inhibitor [JAKi]), from the Rheumatology Outpatient Clinic of the University of Rome Tor Vergata. We monitored the response to treatment for 12 months based on the Disease Activity Index for PsA (DAPsA) score to assess therapeutic efficacy and, consequently, identify subgroups of patient responders and non-responders. Responders were defined based on achievement of low disease activity (DAPSA ≤ 14) or a ≥ 50% improvement from baseline (DAPSA50), in line with the GRAPPA treatment targets [19] and validated by OMERACT [20]. This approach aligns with the treat-to-target strategy recommended by EULAR for PsA [21].

We collected a blood sample for each patient at the beginning of therapy (T0) and after 12 months (T12). In addition, we collected blood samples of 34 age- and sex-matched healthy controls (CTRLs). We selected the 12-month time point (T12) to assess treatment response because it allows sufficient time for b/tsDMARDs to exert their full therapeutic effects and for potential biological changes, such as telomere dynamics, to become detectable. Peripheral blood samples from all subjects have been stored at -80 °C until usage. Demographic and clinical features of both groups are reported in Table 1.

**Table 1:** Clinical Characteristics of the cohort

	PsA	CTRLs	*P*-Value
N	50	34	
Female	32 (64.0)	19 (55.9)	N.S
Age	50.58 ± 12.39	44.99 ± 15.08	N.S
Disease duration (years)	7.41 ± 6.43		
TJ	7.90 ± 8.77		
SJ	1.06 ± 2.02		
CRP (mg/L)	4.99 ± 6.46		
pVAS	7.12 ± 1.96		
PtGA	7.26 ± 1.94		
DAPsA	23.63 ± 12.06		
Disease activity	9 Low		
	30 Moderate		
	11 High		
bDMARDs–TNFαi	19 (38.0)		
bDMARDs–IL17Ai	19 (38.0)		
tsDMARDs–JAKi	12 (24.0)		
Concomitant csDMARDs	21 (42.0)		
Onychopathy	11 (22.0)		
Enthesitis	22 (44.0)		
Dactylitis	12 (24.0)		
Psoriasis	35 (70.0)		
Axial PsA	16 (32.0)		
Metabolic comorbidities	35 (70.0)		
BMI	25.6 ± 4.97		

All categorical variables are expressed as number (%) and numerical ones as mean ± SD, unless otherwise specified. P-value was calculated using t-test. N = number; N.S. = not significant; TJ = numbers of tender joints; SJ = numbers of swollen joints; CRP = C-reactive protein; pVAS = patient pain assessment; PtGA = patient global assessment; DAPsA = Disease Activity Index for PsA; bDMARDs = biological disease-modifying antirheumatic drugs, tsDMARDs = target synthetic disease-modifying antirheumatic drugs; TNFαi: Tumor Necrosis Factor α inhibitor; IL17i: interleukin 17A inhibitor; JAKi: Janus kinase inhibitor; csDMARDs: Conventional synthetic disease-modifying antirheumatic drugs; BMI: Body Mass Index.

All subjects provided informed consent. The study protocol was approved by the local ethics committee (Approval No. RS186/16, November 9th 2016) of the Policlinico Tor Vergata in Rome, Italy.

### Telomere length and mtDNA copy number evaluation

We extracted nuclear and mitochondrial DNA from whole blood samples using EZ1 DNA Blood Kit. We evaluated the relative TL adapting the protocol described by Cawthon [22]. In particular, we performed a quantitative PCR (qPCR) to determine the ratio of telomeric repeat copy signal (TEL) and a reference single-copy gene signal (β-globin [HBB]). The Ct values were concurrently determined in each sample during the same qPCR run. We calculated the relative TL in leukocytes of each subject as T/S ratio (= 2^−(Ct (TEL)−Ct (HBB))^).

Then, we evaluated the mtDNA copy number following the protocol described by Rooney [23]. We quantified the copies using a qPCR with primers amplifying a nuclear DNA region (hemoglobin subunit beta [HGB]) and a mtDNA region (NADH dehydrogenase subunit 1, [ND1]) simultaneously. The Ct values for the nuclear HGB gene and mitochondrial ND1 gene were concurrently determined in each sample during the same qPCR run. We calculated the relative content of mtDNA copy number in the leukocytes of each subject according to the following Eq. 2 × 2 ^(Ct (HGB)−Ct (ND1))^.

We performed all the reactions in triplicate using ABI 7500 Fast Real-time PCR System (Applied Biosystems, Foster City, CA, USA) (SYBR Green Assay, Applied Biosystems). Interassay reproducibility was ensured by including reference samples in each analysis.

### Oxidative damage determination

We evaluated the presence of 8‑hydroxyl 2'‑deoxyguanosine (8‑OHdG), the most common marker of oxidative DNA damage, by modifying the protocol described by Alwehaidah [15]. For each subject, we digested 100 ng of DNA with 1 U of formamidopyrimidine [fapy]‑DNA glycosylase (FPG enzyme) (New England Biolabs, Inc.), and incubated it for 1 h at 37 °C. Next, we performed a qPCR using the digested DNA to evaluate the oxidative damage in the telomere region and in mtDNA following the same conditions described in the previous paragraph. Lastly, we calculated the DNA damage as ΔCt (= Ct treated‑Ct untreated). The FPG enzyme treatment of DNA leads to a decrease in PCR efficiency in presence of the 8‑OHdG and, consequently, to an increase in Ct value.

### Statistical analysis

First, we verified the normal distribution of data using the Kolmogorov–Smirnov test. Accordingly, we compared clinical and demographic variables, TL, mtDNA copy number, and oxidative damage between PsA patients and CTRLs using t-test or Mann–Whitney U test. Instead, for the comparisons between PsA patients at baseline (T0) and at the 12-month follow-up (T12) we used a paired t-test. We assessed potential linear correlation using Pearson correlation analysis. The receiver operating characteristic (ROC) curve was performed to evaluate the ability to discriminate PsA patients from the control group. A *p*-value of ≤ 0.05 was considered significant for all tests. Data are presented as median and interquartile range (IQR). Statistical analyses were performed using SPSS version 19 (IBM Corp., Armonk, NY, USA), and graphs were created using GraphPad Prism 9 (GraphPad Software, USA).

## Results

### Patients clinical characteristics at baseline

Our study cohort included 50 PsA patients and 34 CTRLs, with a prevalence of female participants in both groups (see Table 1). PsA patients had a long-standing disease, with a mean duration of approximately 7.41 ± 6.43 years. They exhibited a moderate disease activity (DAPsA score of 23.63 ± 12.06) and reported an important disease burden as reflected by pain VAS (pVAS) and patient global assessment (PtGA) of 7.12 ± 1.96 and 7.26 ± 1.94, respectively. Clinically, the majority of PsA patients presented psoriasis (70.8%) and nearly half enthesitis (44.2%). Dactylitis and axial involvement were observed in a smaller proportion of the cohort (23.4% and 32.7%, respectively). Regarding therapy, there was a similar distribution between patients starting TNFi (n = 19; 38%) and IL17Ai (n = 19; 38%), while 12 (24%) patients were treated with JAKi.

### Telomere length and oxidative damage

**Fig. 1:**
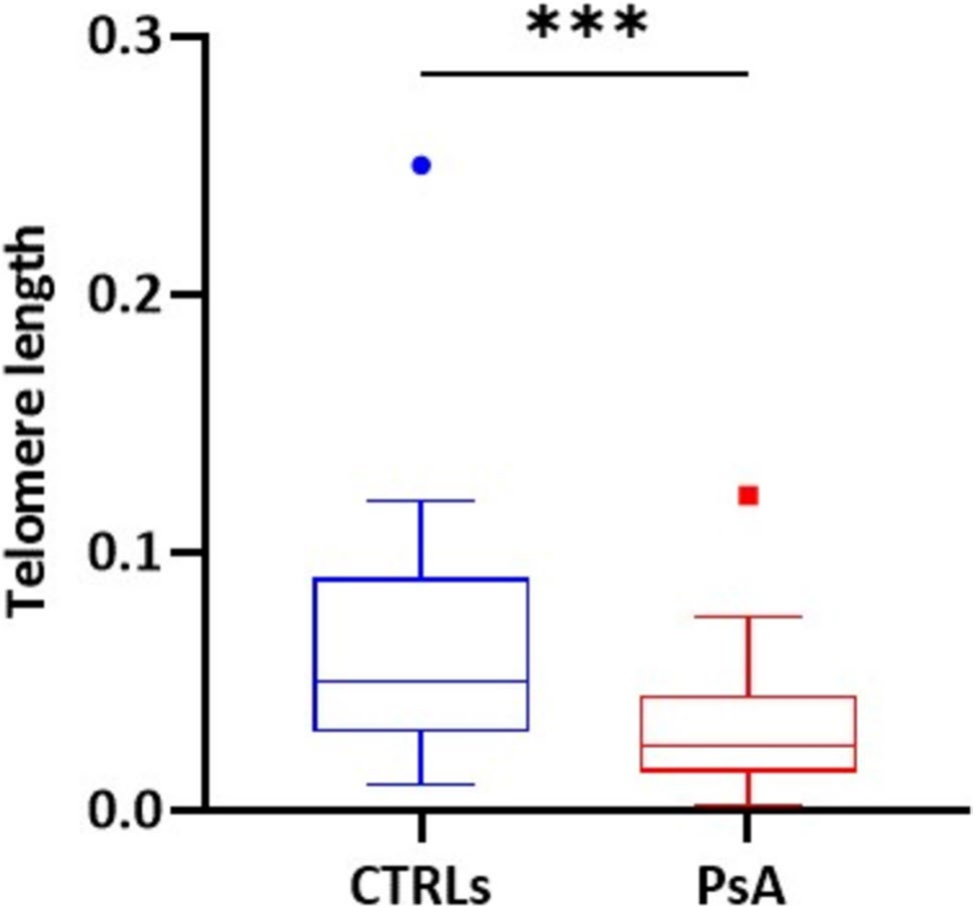
Comparison of relative TL between CTRLs and PsA patients. ***P (Mann–Whitney U test) = 0.005

We evaluated the relative TL of 50 PsA patients at the beginning of therapy (T0) and 34 age- and sex-matched CTRLs. TL was significantly shorter in PsA patients compared to CTRLs (*P* = 0.005). In particular, we observed that the median TL ratio was 0.025 (IQR: 0.015–0.045) in PsA patients compared to 0.050 (IQR: 0.030–0.090) in the control group (Fig. 1).

Furthermore, we assessed oxidative DNA damage in the telomeric region in both PsA patients and CTRLs, but we did not observe any differences between the two groups (data not shown). However, in PsA patients, oxidative telomeric damage positively correlated with disease duration (*P* = 0.042, R = 0.308). Although not statistically significant, we observed an increase in the oxidative telomere damage in patients with disease duration greater than 5 years and in those with moderate/high disease activity, as measured by the DAPsA score (*P* = 0.06). Lastly, we also observed that oxidative damage in the telomeric region positively correlated with body mass index (BMI) (*P* = 0.011, R = 0.418) and was significantly higher in PsA patients with metabolic comorbidities (*P* = 0.025).

### Mitochondrial DNA copy number and oxidative damage

**Fig. 2:**
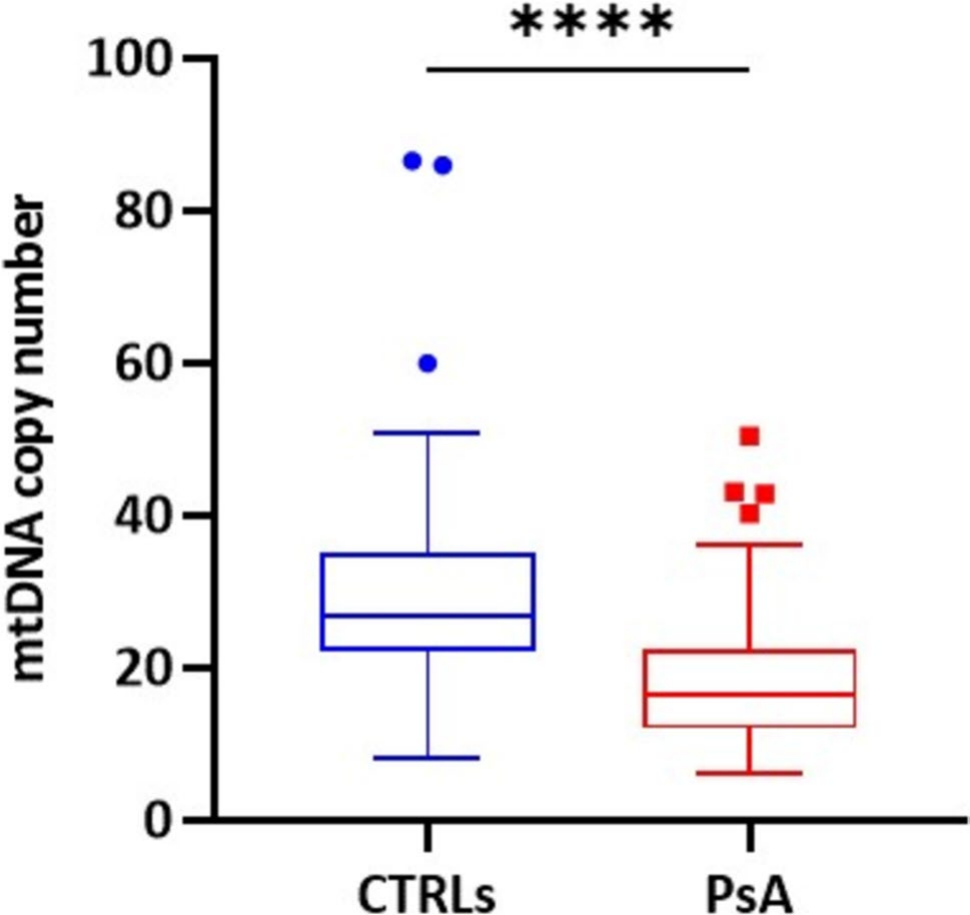
Comparison of mtDNA copy number between CTRLs and PsA patients. ****P (Mann–Whitney U test) < 0.0001

We then evaluated the mtDNA copy number in the same cohorts of patients and controls. PsA patients showed a significantly lower amount of mtDNA copy number compared to CTRLs (*P* < 0.0001). In particular, we observed that the median number of mtDNA copies was 16.43 (IQR: 11.94–22.33) in PsA patients compared to 26.71 (IQR: 22.15–35.04) in the control group (Fig. 2).

In mtDNA, we also estimated the oxidative DNA damage, but we observed no significant difference between these two groups (data not shown). However, in PsA patients, the oxidative mtDNA damage seems to correlate positively with BMI (*P* = 0.007, R = 0.444).

### Patients evaluation after 12-month follow-up

To assess therapeutic efficacy to the three selected classes of drugs, we monitored the treatment response for 12 months. Following, we identified subgroups of patients based on their response to the treatments. Specifically, 36 patients continued to respond (responders), while 14 discontinued the treatment due to lack of efficacy (non-responders). At baseline, we did not observe differences in clinical and serological characteristics or in disease duration between responders and non-responders.

Based on treatment response, we evaluated the relative TL, the mtDNA copy number and the oxidative damage in whole blood samples collected at baseline (T0) from responders and non-responder patients, but we observed no significant difference between these two groups (data not shown).

Lastly, we assessed all these biomarkers of cellular senescence in whole blood samples collected at the 12-month follow-up of the 36 responder PsA patients. As shown in Table 2, after 12 months of therapy, all variables assessed demonstrated a significant improvement. The mean DAPsA score significantly decreased from 22.70 ± 10.35 to 11.81 ± 7.21 (*P* = 3.52E-06), indicating a state of low disease activity following treatment.

**Table 2:** Clinical and serological characteristics of responder patients at T0 and T12

	PsA T0	PsA T12	*P*-Value
N	36	36	
TJ	6.71 ± 7.12	2.12 ± 3.65	0.001
SJ	0.86 ± 1.26	0.32 ± 0.91	0.049
CRP (mg/L)	5.4 ± 7.14	2.22 ± 1.95	0.015
pVAS	7.16 ± 2.07	4.69 ± 2.41	2.26E-05
PtGA	7.39 ± 2.03	4.76 ± 2.10	1.44E-06
DAPsA	22.70 ± 10.35	11.81 ± 7.21	3.52E-06
Disease activity	0 Remission	5 Remission	
	6 Low	20 Low	
	23 Moderate	10 Moderate	
	7 High	1 High	

All categorical variables are expressed as mean ± SD, unless otherwise specified. *N*. Number, *TJ* numbers of tender joints, *SJ* numbers of swollen joints, *CRP* C-reactive protein, *pVAS* patient pain assessment, *PtGA* patient global assessment, *DAPsA* Disease Activity Index for PsA

**Fig. 3:**
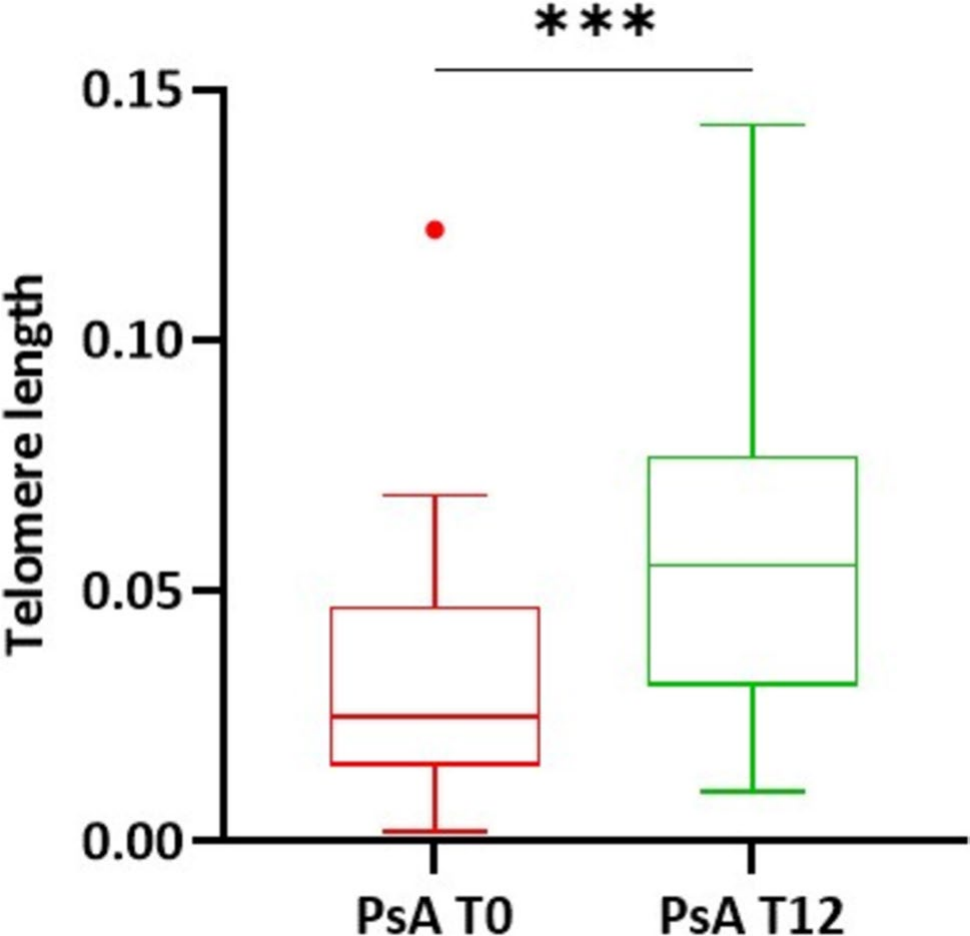
Comparison of relative TL between PsA at T0 and T12 follow-up. ***P (Paired t-test) = 0.0005

**Fig. 4:**
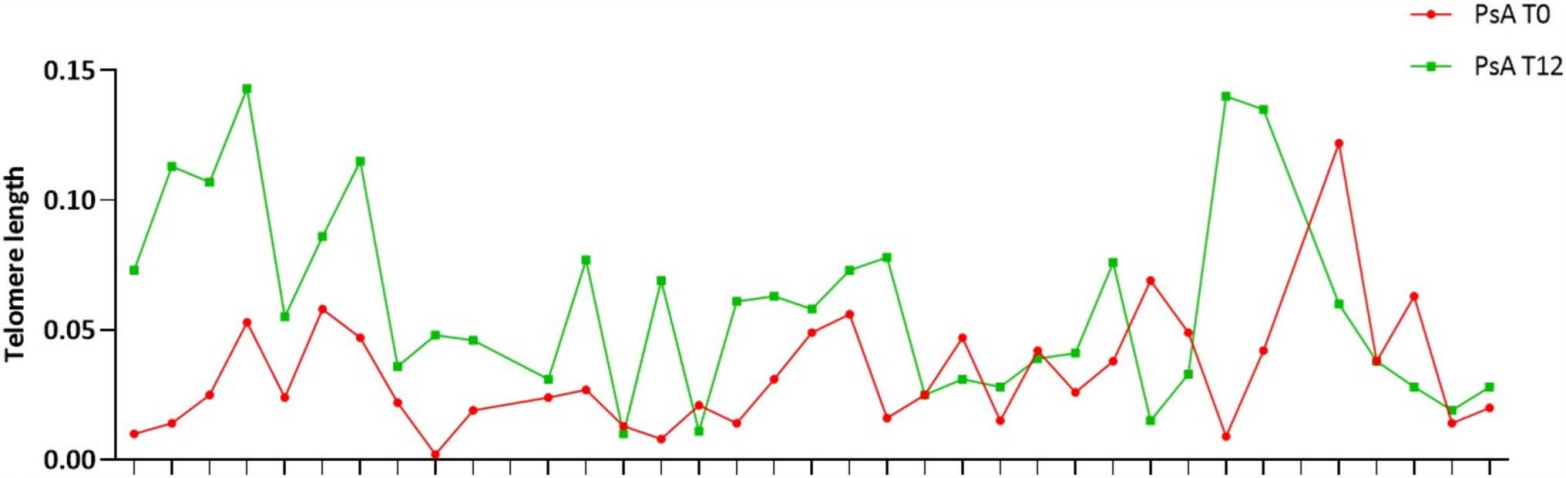
Comparison of TL value at T0 and T12 follow-up for each PsA patient

**Fig. 5:**
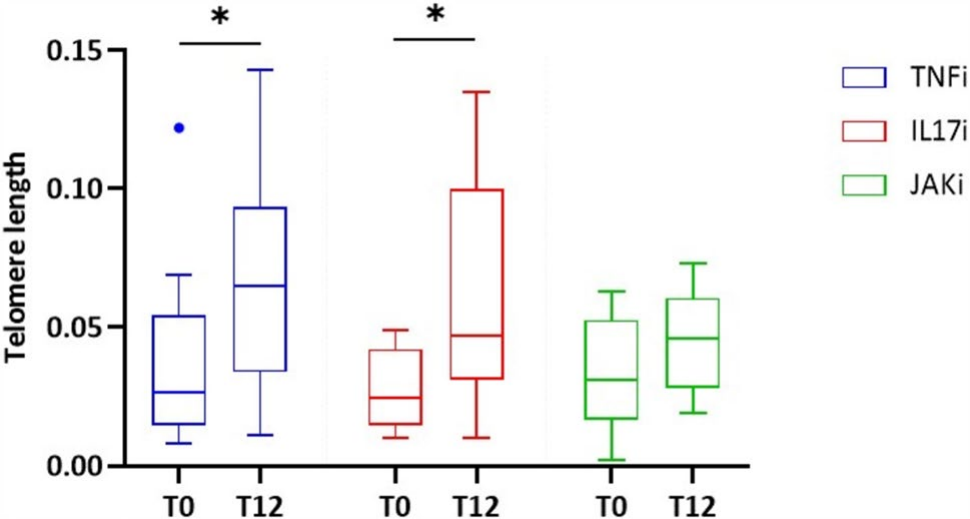
Comparison of relative TL between PsA T0 and PsA T12 patients treated with different class of drugs. *P (Paired t-test) = 0.04; *P (Paired t-test) = 0.02

We observed that, after 12 months, TL was significantly longer compared to the length at T0 (*P* = 0.0009) and reached values comparable to those of the CTRLs group. In fact, we determined that the median TL ratio was 0.055 (IQR: 0.031–0.077) in PsA patients at T12 (Figs. 3 and 4). In Figs. 4 and 5, we reported the TL value at T0 and T12 for each single patient; as shown, it is evident that most patients exhibit an increase in TL value at T12 compared to T0. After patients’ stratification based on the class of drugs, the result is confirmed in patients treated with TNFαi (*P* = 0.04) and IL17Ai (*P* = 0.02) (Fig. 5).

**Fig. 6:**
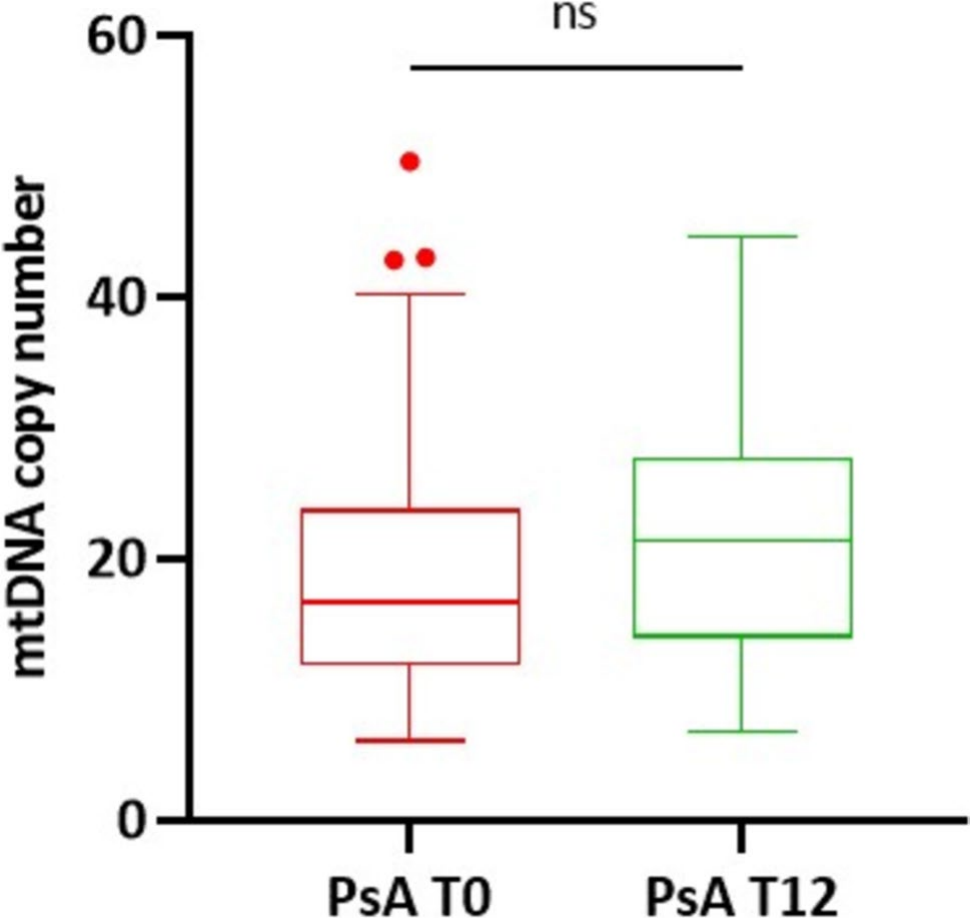
Comparison of mtDNA copy number between PsA at T0 and T12 follow-up. ns (Paired t-test) = not significant

On the contrary, regarding the mtDNA copy number, we observed that the median number did not differ between T0 and T12 in treated PsA patients (Fig. 6).

## Discussion

PsA is a complex rheumatologic disease characterized by chronic inflammation, in which oxidative stress and cellular senescence seem also to play an important role in its pathogenesis [5]. These processes contribute to immune dysregulation and tissue damage, and could provide some potential biomarkers for disease activity and therapeutic response.

The results of our study suggest an important intersection of PsA pathogenetic mechanisms with OS and cellular immunosenescence. In fact, the significant decrease in both TL and mtDNA copy number observed in PsA patients compared to CTRLs indicates an acceleration of cellular aging mechanisms, probably due to the chronic inflammation.

Literature data on other autoimmune rheumatic diseases, such as rheumatoid arthritis and systemic lupus erythematosus, have already described an alteration of the telomere region [7, 10, 24]. Our data are consistent with this evidence; in fact, PsA patients exhibit significantly shorter TL than controls. Interestingly, in our cohort, reduced TL correlates positively with disease duration, suggesting that chronic persistent inflammation contributes progressively to telomere erosion. This observation is further supported by studies showing that inflammatory cytokines such as TNFα and IL17, central in the pathogenesis of PsA, are able to inhibit telomerase activity and promote OS [25, 26].

However, it is known that the mitochondrial integrity is critical for the regulation of innate and adaptive immunity [26]. Recent studies reported that T cell senescence is actively driven by a bidirectional interplay between telomere erosion and mitochondrial dysfunction [27, 28]. In particular, telomere shortening has been strongly associated with impaired mitochondrial biogenesis and reduced mtDNA replication [29]. Accordingly, our results highlighted a significant reduction in mtDNA copy number in PsA patients compared to controls. These data are also consistent with previous studies reported in the literature [15, 30], further supporting the evidence that mitochondrial dysfunction is another key feature of immune-mediated and chronic inflammatory diseases.

Although oxidative damage in the telomere region and mtDNA was not significantly different between PsA patients and controls, a positive correlation with BMI and the presence of metabolic comorbidities emerged. PsA is knowingly linked to metabolic comorbidities such as dyslipidemia, insulin resistance, and obesity, especially in male patients. These metabolic derangements are not passive consequences of chronic inflammation but actively contribute to immune dysregulation and sustain a pro-inflammatory environment, driving disease progression in PsA through shared pathways linking systemic inflammation and metabolic dysfunction, atherosclerosis, and OS [31, 32]. Obesity and metabolic syndrome are known to interact with the immune system, stimulating the production of pro-inflammatory cytokines, including TNFα and IL17A [33], with a potential synergistic role in promoting oxidative damage and telomere instability [9]. Moreover, the positive correlation of telomeric oxidative damage with disease duration suggests that telomeric damage in PsA might emerge gradually over time, concurrently to the biological processes of accelerated cellular aging and immune dysregulation described in chronic inflammatory disease [1].

Notably, our study also shows that treatment with bDMARDs, particularly TNFαi and IL17Ai, is associated with a significant increase in TL after 12 months of treatment, reaching median values comparable to those of controls. We could hypothesize that an effective control of both inflammatory response and disease activity may have a positive impact on genomic stability, potentially slowing down the processes of cellular senescence [18]. In contrast, mtDNA copy number did not change significantly after treatment, suggesting that mitochondrial dysfunction might require more time to reverse or be less invertible than nuclear damage. This finding highlights the need for further longitudinal studies to define the dynamics of mitochondrial stability during therapy.

Despite the promising results, this study has some limitations. The relatively small sample size, particularly in the treatment subgroups, could limit the detection of possible correlations. Larger cohorts and long-term follow-up will be required to confirm these findings and to fully understand the impact of biological therapy on cellular senescence processes. In addition, it will also be interesting to replicate the results on immune cell subpopulations.

## Conclusions

In conclusion, this study provides novel insights for the molecular mechanism underlying PsA pathogenesis, suggesting the potential use of TL and mtDNA copy number as biomarkers for assessing cellular senescence in PsA. Our results also show that the increase in cellular senescence biomarkers in PsA patients seems to be partially mitigated by bDMARDs therapy, particularly in terms of telomeric shortening. If confirmed, these results further support the potential use of TL as biomarker for monitoring therapeutic efficacy.

## Data Availability

No datasets were generated or analysed during the current study.
